# Supplementary material for: HIVprotI: an integrated web based platform for prediction and design of HIV proteins inhibitors
Source: J Cheminform. 2018 Mar 9;10:12. doi: 10.1186/s13321-018-0266-y (PMC5845081; doi:10.1186/s13321-018-0266-y)
Supplement: Supplementary file 2 — Additional file 2. Source code of HIVProtI web server. [file 13321_2018_266_MOESM2_ESM.zip › HIVprotI_Source-code/hivprotI/weka-3-6-6/doc/weka/gui/beans/ClassValuePickerBeanInfo.html]

ClassValuePickerBeanInfo


---


|  |  |  |
| --- | --- | --- |
| **Overview** \| **Package** \| **Class** \| **Tree** \| **Deprecated** \| **Index** \| **Help** \| **Weka's home** | |  |
| **PREV CLASS**   **NEXT CLASS** | **FRAMES**    **NO FRAMES**     **All Classes** |  |
| SUMMARY: NESTED \| FIELD \| CONSTR \| METHOD | DETAIL: FIELD \| CONSTR \| METHOD |  |


---


## weka.gui.beans Class ClassValuePickerBeanInfo

```
java.lang.Object
  java.beans.SimpleBeanInfo
      weka.gui.beans.ClassValuePickerBeanInfo
```

**All Implemented Interfaces:**: java.beans.BeanInfo

---

``` public class ClassValuePickerBeanInfo extends java.beans.SimpleBeanInfo ```

BeanInfo class for the class value picker bean

**Version:**
:   $Revision: 7439 $

**Author:**
:   Mark Hall

---

**Field Summary**

**Fields inherited from interface java.beans.BeanInfo**

`ICON_COLOR_16x16, ICON_COLOR_32x32, ICON_MONO_16x16, ICON_MONO_32x32`


**Constructor Summary**

`ClassValuePickerBeanInfo()`


| **Method Summary** | |
| --- | --- |
| `java.beans.BeanDescriptor` | `getBeanDescriptor()` |
| `java.beans.EventSetDescriptor[]` | `getEventSetDescriptors()`             Returns the event set descriptors |
| `java.beans.PropertyDescriptor[]` | `getPropertyDescriptors()`             Returns the property descriptors |

**Methods inherited from class java.beans.SimpleBeanInfo**

`getAdditionalBeanInfo, getDefaultEventIndex, getDefaultPropertyIndex, getIcon, getMethodDescriptors, loadImage`

**Methods inherited from class java.lang.Object**

`equals, getClass, hashCode, notify, notifyAll, toString, wait, wait, wait`

**Constructor Detail**

### ClassValuePickerBeanInfo

```
public ClassValuePickerBeanInfo()
```


**Method Detail**

### getEventSetDescriptors

```
public java.beans.EventSetDescriptor[] getEventSetDescriptors()
```

:   Returns the event set descriptors

    :   **Specified by:**: `getEventSetDescriptors` in interface `java.beans.BeanInfo` **Overrides:**: `getEventSetDescriptors` in class `java.beans.SimpleBeanInfo`
    :   **Returns:**: an `EventSetDescriptor[]` value

---


### getPropertyDescriptors

```
public java.beans.PropertyDescriptor[] getPropertyDescriptors()
```

:   Returns the property descriptors

    :   **Specified by:**: `getPropertyDescriptors` in interface `java.beans.BeanInfo` **Overrides:**: `getPropertyDescriptors` in class `java.beans.SimpleBeanInfo`
    :   **Returns:**: a `PropertyDescriptor[]` value

---


### getBeanDescriptor

```
public java.beans.BeanDescriptor getBeanDescriptor()
```

:   **Specified by:**: `getBeanDescriptor` in interface `java.beans.BeanInfo` **Overrides:**: `getBeanDescriptor` in class `java.beans.SimpleBeanInfo`


---


|  |  |  |
| --- | --- | --- |
| **Overview** \| **Package** \| **Class** \| **Tree** \| **Deprecated** \| **Index** \| **Help** \| **Weka's home** | |  |
| **PREV CLASS**   **NEXT CLASS** | **FRAMES**    **NO FRAMES**     **All Classes** |  |
| SUMMARY: NESTED \| FIELD \| CONSTR \| METHOD | DETAIL: FIELD \| CONSTR \| METHOD |  |


---
